# The “double-edged sword” effect of shared leadership on employee voice behavior

**DOI:** 10.3389/fpsyg.2025.1560554

**Published:** 2025-05-23

**Authors:** Yina Bai, Haoran He

**Affiliations:** School of Business Administration, Liaoning Technical University, Huludao, China

**Keywords:** shared leadership, voice behavior, double-edged sword, empowerment pathway, disempowerment pathway

## Abstract

**Introduction:**

This study investigates the dual-path effects of shared leadership on employee voice behavior through the Empowerment-Servitude Model, with a focus on uncovering the underlying psychological mechanisms. Additionally, it identifies key organizational and individual factors influencing employee voice behavior, offering theoretical insights for strategies aimed at behavioral enhancement.

**Methods:**

This study adopted a multi-method approach, integrating online questionnaires and on-site enterprise visits across two time phases. Data collection yielded 624 valid responses from 13 corporations in Dalian, which were subsequently analyzed statistically.

**Results:**

The results suggest that, on the one hand, shared leadership, as an empowerment mechanism, positively influences employee voice behaviors through the empowerment pathway associated with organizational status perception. On the other hand, shared leadership, as a depletion mechanism, negatively impacts employee voice behaviors via the servitude pathway, characterized by emotional exhaustion. Employee empowerment expectations play a critical role in triggering these contrasting mechanisms. Specifically, higher levels of employee empowerment expectations mitigate the negative impact of the depletion mechanism while enhancing the positive effects of the empowerment mechanism.

**Discussion:**

This study makes three key contributions to the literature: first, it advances a nuanced understanding of the relationship between shared leadership and employee voice behavior. Second, by examining the moderating role of employee empowerment expectations, it elucidates boundary conditions influencing this relationship. Third, the findings underscore the criticality of empowerment expectations in organizational practice, suggesting that managers implementing shared leadership should systematically assess employees' subjective empowerment expectations, and leverage these expectations to enhance the model's positive effects on voice behaviors.

## 1 Introduction

Voice behavior refers to employees' proactive and spontaneous efforts to express work-related opinions or suggestions in order to solve problems within the organization. Scholars have identified this behavior as key to improving decision-making quality, reducing organizational risks and enhancing organizational performance (Detert and Burris, 2007). It has also become a prominent topic in organizational behavior research in recent years. Related studies have shown that employees' willingness to offer suggestions largely depends on the leadership style (Xin et al., 2021). Compared to vertical leadership, which focuses on formal delegation, the shared leadership model—emphasizing the rotation and sharing of leadership roles within teams and informal delegation—more effectively stimulates employees' sense of participation, promotes knowledge exchange within the organization and supports the achievement of organizational goals (Klasmeier and Rowold, 2022). A substantial body of research has concluded that shared leadership profoundly influences employees' constructive behaviors. For instance, Zhang et al. (2020) argued that shared leadership enhances employees' perception of power, which positively affects both facilitative and inhibitory behaviors. Yang and Chen (2020) suggested that shared leadership positively influences constructive employee behavior through psychological ownership. However, some studies have shown that shared leadership may negatively impact employees' constructive behavior. Liu (2017) contended that high levels of shared leadership increase employees' perception of interpersonal risk, thereby reducing organizational citizenship behaviors, such as voice. Zhao (2022) further argued that shared leadership inhibits employees' desire to express themselves due to fear of negative evaluation, leading to silence. Base on the Demands-Resources (JD-R) theory (Bakker and Demerouti, 2007), Shared leadership plays a dual role of job resources and job demands, and has a differentiated impact on employees. On one hand, it provides employees with resources such as greater decision-making autonomy and opportunities for authentic self-expression; on the other hand, it imposes additional demands through heightened responsibilities and task burdens.

This study constructs a dual-mediation model, based on the Empowerment Enslavement Paradox and the Conservation of Resources Theory, to integrate the positive and negative impacts of shared leadership on employees' voice behaviors. The model consists of two paths: an “empowerment” path, where power-sharing by leaders increases employees' perceived organizational support, and an “enslavement” path, where power rotation and responsibility-sharing by leaders lead to emotional exhaustion. In the empowerment path, leadership roles under the shared leadership model are assumed by employees who hold heterogeneous resources. Employees granted power due to their specific resources influence the organization and experience support and respect from both the organization and other members, which fosters a positive perception of shared leadership and stimulates voice behavior. In the enslavement path, the shared leadership model emphasizes the selection of employees as leaders based on specific tasks and situations. Individuals with heterogeneous knowledge within the organization have the potential to become leaders, and the rotation of power brings additional responsibilities and work pressure, resulting in emotional exhaustion. This negative perception of shared leadership leads to a reduction in constructive behaviors.

As organizations face increasing uncertainty and pressure from industrial transformation and upgrading, leaders can no longer independently address all challenges. It is crucial for organizations to leverage employees' proactive perspectives and ideas to continuously improve (Li et al., 2024). Conversely, employee silence on work-related issues often stifles organizational vitality and leads to missed opportunities. Thus, exploring the conditions under which employees are motivated to speak up is essential (Morrison and Milliken, 2000). Previous studies have mainly explored how organizational-level factors motivate employees to offer advice, while overlooking the role of “expectation,” which reflects individual self-motivation (Yin et al., 2021; Wang and Duan, 2021). Employee empowerment expectations are an extension of the concept of expectations in the empowerment framework, affecting how employees assume leadership roles within the organization (Labianca et al., 2000). Therefore, it is argued that individual employees with different levels of empowerment expectations have varying perceptions of the shared leadership model, which in turn impacts their constructive behaviors. This paper introduces “employee empowerment expectancy,” a trait variable reflecting self-motivation, to explore its moderating role in the “empowerment” and “enslavement” paths of shared leadership's influence on voice behavior.

## 2 Theoretical background and hypothesis development

### 2.1 The relationship between shared leadership and employee voice behavior

The shared leadership model emphasizes the distribution of leadership roles within a team, where team members engage in mutual leadership and influence through power sharing, ultimately contributing to team achievements and organizational goals (Pearce and Conger, 2002). This model not only improves the formal authority found in vertical leadership by moving away from the hierarchical superior-subordinate relationship but also fosters a cooperative, win-win team environment (Zhao et al., 2018). According to Liu (2012), the core features of shared leadership include leadership power sharing, interdependence of leadership behaviors, and the opening of leadership boundaries. Specifically, the shared leadership model represents a dynamic process in which leadership roles are continuously rotated among team members based on the demands of the task at hand. In this model, power, leadership responsibilities, and decision-making are shared across the team.

After Dyne and LePine (1998) pioneered the conceptualization of constructive behavior within organizational behavior research, defining it as a prototypical form of organizational citizenship behavior characterized by cooperative-motivated expressions of work-related information, ideas, and opinions, this construct has garnered increasing scholarly attention. Extant literature generally addresses three key aspects of voice behavior: first, scholars conceptualize voice behavior as intentional, change-oriented organizational behaviors that enhance organizational effectiveness while mitigating systemic risks (Morrison, 2011; Ng and Feldman, 2012). Second, constructive voice may target task-related or interpersonal issues, often entailing interpersonal risks due to its challenging nature (Morrison, 2011). Third, such behavior frequently transcends prescribed job duties, reflecting discretionary extra-role contributions (Lu et al., 2020; Xin et al., 2021). The shared leadership model, by flattening hierarchical boundaries, enables employees to perceive greater voice and leadership authority in their domains of expertise. This structural empowerment fosters problem identification and facilitates constructive voice behaviors (Zhao et al., 2018; Yang and Chen, 2020).

However, the effectiveness of shared leadership is contingent upon specific team contexts (Jian and Chen, 2023). Employees' technical maturity and knowledge are crucial factors for the successful implementation of shared leadership (Hoch and Dulebohn, 2017; Scott-Young et al., 2019). The empowerment-slavery paradox suggests that workplace technology embodies both “empowerment” and “enslavement” (Jarvenpaa and Lang, 2005). On the one hand, technological maturity and knowledge ownership empower employees to equally share leadership responsibilities. This empowerment fosters a sense of ownership, encouraging employees to take responsibility as part of their organizational identity or work content and actively engage in contribution. On the other hand, in teams with shared leadership, employees—especially those with high levels of technical expertise or knowledge—may face additional work and leadership responsibilities, creating the paradox of “the more capable you are, the more work you are assigned.” According to the theory of resource conservation, when employees take on more work and leadership responsibilities and are under pressure from both work demands and interpersonal relationships, they may reduce their input into innovative thinking and work. This leads to a decrease in out-of-role behaviors to conserve their limited time and energy, thus inhibiting their constructive behaviors. In summary, the shared leadership model acts as a double-edged sword, triggering different employee behaviors. From an empowerment perspective, shared leadership's power-sharing positively influences employee voice behaviors. However, from the perspective of “enslavement,” the increased responsibility imposed by shared leadership can create burdens that negatively affect voice behaviors. Given the dual influence of shared leadership on employee voice, this study does not assume a simple one-way relationship.

### 2.2 Empowerment pathways: the mediating role of perceived organizational status

Perceived organizational status reflects employees' subjective evaluation and cognitive judgment of their positional standing within an organization (Seeman and Berkman, 1988). Empirical evidence suggests that leadership serves as a critical source of status-related information, with leader behaviors enhancing employees' perceived insider status and organization-based self-esteem (Wu and Zheng, 2018). Diverging from conventional leadership paradigms, shared leadership embodies a bottom-up emergent process that grants team members substantial autonomy. Within this framework, individuals may assume leadership roles by leveraging their unique resource advantages, including specialized knowledge, technical expertise, or social competencies. Particularly in knowledge-intensive teams, members possessing superior technical skills and task-relevant capabilities naturally accrue leadership authority and responsibilities, enabling them to autonomously address challenges through their knowledge and potential. Research demonstrates that increased decision-making autonomy and organizational influence positively correlate with enhanced perceived organizational status (Eisenberger et al., 2002). The distinctive empowerment characteristic of shared leadership fulfills employees' psychological needs for self-worth and activates self-efficacy mechanisms, thereby cultivating elevated status perceptions. As a relational asset rooted in interpersonal dynamics (Yin et al., 2021), status perception flourishes within shared leadership contexts through their inherent emphasis on fostering harmonious, egalitarian relationships characterized by mutual support and care (Liao et al., 2016). Employees perceive these high-quality relationships as valuable resources. Grounded in social exchange theory, when individuals experience trust, support, understanding, encouragement, and respect from team members, their psychological ownership becomes activated, subsequently augmenting organizational status perceptions. This study proposed that shared leadership promotes perceived organizational status.

**Hypothesis 1:** Shared leadership promotes perceived organizational status.

In previous studies, scholars generally agree that perceived organizational status affects employees' psychological, emotional, and social cognition, and directly influences their work styles and attitudes (Fuller et al., 2007). Moreover, the motivation and behavior of employees depend on how valid and secure they perceive these constructs (Morrison, 2011). On one hand, when employees perceive higher organizational support and prestige, they are more likely to believe they hold a higher position within the organization. This belief fosters a sense of responsibility and obligation, which, in turn, enhances their motivation and ability to exert influence. In return, employees are more likely to respond to organizational issues and offer advice and suggestions for improvement (Amabile, 1988; Luo et al., 2017). On the other hand, according to the theory of resource conservation, employees with a high perception of organizational status—who are respected, supported, and praised—tend to exhibit greater self-confidence, optimism, and positivity, enhancing their psychological capital. This boosts their belief that they will continue to be treated favorably, fostering greater trust in the organization (Berger et al., 2014). As a result, employees become less focused on the risks and resource losses associated with constructive behavior, thereby contributing more effectively to the organization's development. In summary, while voice is an extra-role behavior not explicitly required by the job, it involves multiple uncertainties, such as interpersonal challenges. Moreover, influenced by the traditional Chinese concept of “mediocrity,” many individuals possess a lower appetite for risk and are reluctant to advocate in the workplace (Zhou and Liao, 2012). However, from the perspective of the empowerment pathway in empowerment-slavery theory, teams that implement shared leadership no longer rely solely on the command of a single leader. Through empowerment, team members gain more autonomy in decision-making and perceive a higher status within the organization. This, in turn, reduces concerns about being blamed by colleagues when proposing new solutions, thereby stimulating a stronger sense of service to the organization and a greater willingness to challenge the status quo. This empowerment mechanism, triggered by the shared leadership model, leads to more proactive and constructive behavior aimed at organizational change. So, the following hypothesis is predicted:

**Hypothesis 2:** the promotion of perceived organizational status by shared leadership can affect employee voice behavior, thus perceived organizational status positively mediating the link between Shared leadership and employee voice behavior.

### 2.3 Disempowerment pathways: the mediating role of emotional exhaustion

Emotional exhaustion refers to a state of fatigue resulting from the overuse of mental and emotional resources caused by workplace stressors, and is considered an outcome of stress responses (Maslach et al., 2001). Previous studies have indicated that leadership models, such as transformational leadership (Green et al., 2013) and authentic leadership (Kampa et al., 2017), which involve care, encouragement, and support, can effectively alleviate emotional exhaustion in employees. However, some studies have suggested that positive leadership models, such as ethical leadership (Lee et al., 2021), transformational leadership (Zwingmann et al., 2016), and empowering leadership (Wang Z. et al., 2019), can, paradoxically, exacerbate emotional exhaustion in employees. Advising employees can be seen as a strategic process, where employees weigh the potential benefits and risks before offering advice (Wang et al., 2020). According to resource conservation theory, individuals are motivated to “conserve resources” and “acquire resources.” When employees face threats to their resources, experience mismatches between input and reward, or suffer from overconsumption, they are likely to have stress reactions, which in turn lead to emotional changes (Hobfoll, 1989, 2001). The shared leadership model promotes high levels of empowerment, but when this empowerment reaches its limits, it can create workplace stress, depleting individual resources and leading to emotional exhaustion.

In teams implementing the shared leadership model, members with higher skill levels and knowledge necessary for solving team tasks are empowered to take on leadership roles. As a result, they assume greater leadership responsibilities and work tasks. However, the increasing workload and task complexity not only encroach upon the employees' attention resources, which should be devoted to their own tasks, but also introduce additional cognitive labor, resulting in cognitive overload. Furthermore, in the shared leadership model, employees alternate between roles as both “followers” and “leaders.” This constant role-switching depletes their energy, time, and other resources. The shared leadership model places a strong emphasis on team members' ability to collaborate effectively to achieve shared goals. Additionally, the model encourages interactions between team members to enhance collective intelligence and achieve organizational objectives through group discussions or team interactions (Yang and Chen, 2020). Employees in leadership roles are also entrusted with the responsibility of assisting other members, facilitating team exchanges, and mediating opposing viewpoints when necessary (Zhang et al., 2020). These interactions may impact the vested interests of other team members, leading to a depletion of the individual's interpersonal resources. Based on the above analysis, this study concludes that the shared leadership model triggers the depletion of employees' resources, including attention, personal energy, and time, which ultimately leads to emotional exhaustion. So, the following hypothesis is predicted:

**Hypothesis 3:** shared leadership increases employee emotional exhaustion.

Emotional exhaustion refers to a state in which an individual lacks vitality and their emotional resources are near depletion. This condition can trigger negative work behaviors in employees, such as job neglect (Greenbaum et al., 2014), turnover (Wright and Cropanzano, 1998), silence (Yi et al., 2018), and service disruptions (Edmondson et al., 2019), among others. Emotional state directly influences employees' constructive behaviors at work. Positive emotions facilitate constructive behaviors (Fu et al., 2012), while negative emotions inhibit them (Ng and Feldman, 2012). According to resource conservation theory, because individuals have limited resources, they adopt protective mechanisms to preserve or conceal the resources they value in order to prevent damage (Lee et al., 2021; Guo et al., 2024). In situations where interpersonal, cognitive, and psychological resources are overly depleted due to emotional exhaustion, employees tend to adopt defensive measures to safeguard their remaining resources and maintain necessary work status (Van Jaarsveld et al., 2021). The core features of the shared leadership model, such as shared responsibility, joint decision-making, and high empowerment, can lead to excessive stress and perceived resource depletion among employees, which can trigger emotional exhaustion. Therefore, under the shared leadership model, employees may focus more on their own tasks, adopt conservative strategies to ensure their “safety”, and reduce out-of-role behaviors to avoid interpersonal risks. As a result, the degree of employee voice may decrease. So, the following hypothesis is predicted:

**Hypothesis 4:** emotional exhaustion, as a consequence of shared leadership, could reduce employee voice behavior, with Emotional exhaustion serving as negative mediator in the shared leadership-employee voice behavior nexus.

### 2.4 The moderating role of empowerment expectations

Voice behavior is an out-of-role interpersonal communication behavior where employees attempt to change the status quo of their work or organization. It is inherently challenging, and if unsuccessful, may face opposition or rejection from others within the team (Wang S. H. et al., 2019). Given its challenging nature, voice requires strong motivation. Previous studies have primarily explored the motivation behind voice in terms of external factors such as personality traits (Yin et al., 2021), organizational climate (Frazier and Bowler, 2012), and job/task characteristics (Dedahanov et al., 2019). However, the positive role of an individual's self-concept in the context of voice has often been overlooked, as has the fundamental psychological function of “expectations” (Zhang et al., 2016).

Employee empowerment expectations refer to the normative perceptions employees form regarding the duties and obligations of leaders in delegating authority within organizations (Humborstad and Kuvaas, 2013). Wong and Giessner (2018) stated that employee empowerment expectations, as cognitive schemas, define the behaviors leaders should exhibit in their relationships with employees and influence how subordinates assume their job roles relative to their superiors. In team tasks, employees construct different degrees of expectations about the team's empowerment behaviors (Hinkin and Schriesheim, 2008; Yin et al., 2021). The alignment between these expectations and the actual empowerment behaviors of the team affects how employees evaluate their leaders' empowerment behaviors, which in turn influences leadership effectiveness.

When employees' empowerment expectations are high and aligned with the team's empowerment practices, the shared leadership model's features—such as power sharing, responsibility sharing, and joint decision-making—stimulate employees' self-worth and initiative. Moreover, when formal leaders recognize high employee empowerment expectations, they are more likely to grant employees greater autonomy and influence, which enhances their perception of organizational status and encourages them to express work-related ideas freely. In this way, employee empowerment expectations strengthen the shared leadership model's effect on perceptions of organizational status. So, the following hypothesis is predicted:

**Hypothesis 5:** the presence of Employee empowerment expectations intensifies the beneficial link between shared leadership and perceived organizational status. As Employee empowerment expectations escalates, so does the strength of this positive interconnection.

This study suggests that employee empowerment expectations mitigate the attrition mechanism of shared leadership for emotional exhaustion. According to resource conservation theory, employees with high empowerment expectations have a strong willingness to engage in organizational activities. They view “empowerment” as a valuable resource and reciprocate by exhibiting extra-role behaviors and positive performance, which benefit the organization. These employees aim to secure higher levels of empowerment by contributing behaviors that are advantageous to the organization while minimizing behaviors that may harm organizational objectives. Additionally, the acquisition of the valuable resource of “empowerment” reduces their fear of interpersonal and cognitive resource depletion, encouraging them to overlook resource depletion and actively offer positive suggestions. In contrast, employees with low empowerment expectations are likely to resist leadership empowerment, negatively evaluate overloaded tasks, and perceive extra-role behaviors as resource-draining. These employees believe that engaging in such behaviors not only depletes their resources but also limits their ability to acquire and accumulate new resources, placing them in a state of resource loss. When these concerns intensify or when employees lack confidence in managing these challenges, they are more likely to adopt a passive approach, such as waiting or remaining silent (Zhang et al., 2016). So, the following hypothesis is predicted;

**Hypothesis 6:** empowerment expectations negatively moderates the adversarial interaction between shared leadership and employee emotional exhaustion. As Empowerment expectations escalates, the unfavorable link between these elements diminishes.

### 2.5 An integrative moderated mediation model

**Figure 1:**
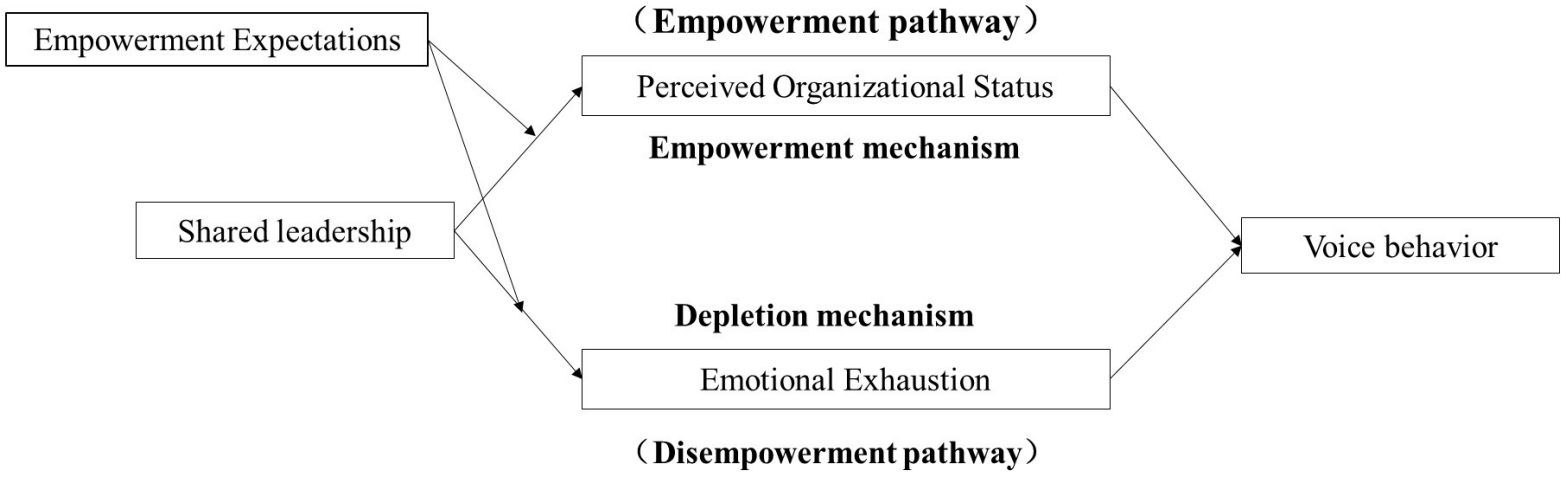
Theoretical model.

Building upon the aforementioned hypotheses, we propose an integrated moderated mediation model (See Figure 1). The increased levels of SL are expected to enhance employees' FWA, consequently benefiting their voice Behavior. However, SL may simultaneously contribute to elevated emotional exhaustion (EE), potentially undermining employees' proactive suggestion-making. When employees' authorization expectations align with leaders' empowering behaviors, they demonstrate greater willingness to internalize decision-making responsibilities, incorporate these into their work routines, and consequently strengthen the positive association between SL and POR (Labianca et al., 2000). Furthermore, employees' expected level of authorization serves as a positive moderator in the relationship between SL and emotional exhaustion, ultimately enhancing their sense of organizational participation. Consequently, we propose the following integrated moderated mediation model:

**Hypothesis 7:** SLP have positive effects on employee voice behavior through increased POS, while SL also have potential negative effects on employee Voice behavior through increased employee emotional exhaustion. The first link of this mediation process will be moderated by Empowerment expectations, such that under conditions of high Empowerment expectations, the positive indirect effects will be stronger, and the negative indirect effect of SLP on employee emotional exhaustion will be mitigated. These combined effects ultimately contribute to improved employee Voice behavior.

## 3 Methodology

### 3.1 Participants and procedure

To ensure that the survey sample matches the shared leadership model, we confirmed that the selected companies and their members met the following three criteria: (1) Participants' work content is interdependent among team members;(2) Participants can assume leadership roles in various areas based on work dimensions; and (3) Participants have a leader responsible for team performance (Chiu et al., 2016; Lyndon et al., 2020).

To ensure sample randomness and representativeness, this study employed a qualified convenience sampling approach within Dalian, China. Among the thirteen selected companies, five were state-owned enterprises, three were European or American multinational corporations, and the remaining four were privately owned. These firms spanned six distinct industries: internet technology, finance, consulting, food processing, real estate, and garment manufacturing. The decision to conduct this survey in Dalian was based on two key considerations. First, as a major port city in Northeast China, Dalian exhibits a high level of economic openness. It hosts not only large state-owned manufacturing enterprises but also a significant presence of foreign-owned firms specializing in international trade, logistics, and production, resulting in considerable diversity in leadership styles. Second, Dalian's industrial structure encompasses a broad spectrum of sectors, including equipment manufacturing, software and information technology, petrochemicals, and fisheries. Consequently, leadership demands vary substantially across these industries, making the city an ideal setting for examining contextual differences in leadership practices.

The survey was conducted in three sequential phases. In the initial phase, the research team identified potential target enterprises through multiple channels, including MBA program networks, professional training seminars, alumni associations, and personal connections. Researchers then engaged in comprehensive consultations with human resource managers or team leaders (e.g., project or department heads) from these enterprises via either on-site visits or telephone interviews. During these interactions, the study's objectives and significance were clearly communicated, and organizational support was secured to facilitate project implementation. In the second stage, we visited the surveyed enterprises and distributed questionnaires to 800 employees who met the above conditions in the form of on-site questionnaires. To ensure anonymity and minimize potential common method biases, we collected the last four digits of each subject's mobile phone number as its unique code in the study to facilitate data collection over two distinct intervals. The survey instrument comprised two key components: (1) demographic information (including age, gender, and educational attainment), and (2) standardized measures assessing shared leadership, organizational status perception, emotional exhaustion, and employee empowerment expectations. A total of 720 employees completed the questionnaire. Approximately 1 month after the initial data collection, we administered a follow-up survey to the same participants to evaluate their perceived voice behavior. From the original sample of 720 respondents, we obtained 624 completed questionnaires at second questionnaire survey, representing an 78% response rate. The final sample consisted of 54.2% male and 45.8% female participants. In terms of age distribution: 4% were under 25 years, 28.4% were 25-34 years, 59.1% were 35–44 years, and 6.1% were 45 years or older. Regarding educational attainment, 49.7% of respondents held a bachelor's degree or higher qualification.

### 3.2 Measurements

This study operationalized the key constructs—including shared leadership, perceived organizational status, emotional exhaustion, empowerment expectations, and employee voice behavior—using validated measurement scales. The original English versions of these instruments underwent rigorous translation procedures, including forward-translation and back-translation, to ensure conceptual equivalence in Chinese. All measures, with the exception of control variables, employed a 5-point Likert-type response format ranging from 1 (“strongly disagree”) to 5 (“strongly agree”). Reliability analyses confirmed that all scales demonstrated satisfactory internal consistency, with Cronbach's alpha coefficients exceeding conventional thresholds for scale reliability.

**Shared leadership (SL)**: the four-dimensional shared leadership scale, which includes “performance expectations, mutual collaboration, power and responsibility sharing, and team learning,” was developed by Liu (2009) and Zhao and Zhao (2012) and used as the foundation for this study. Seventeen items were selected for measurement, including “Our team (department) members and leaders can share power,” which aligns with the characteristics of the knowledge-based teams in this study. The Cronbach's alpha coefficient for this scale was 0.934.

**Perceived organizational status (POR):** this variable was measured using a one-dimensional scale developed by Eisenberger et al. (2002). Four items from this scale were selected to match the present study, including “My organization has a high level of respect for me as an individual,” “My organization consults me when making decisions,” and “My organization asks for my opinions and suggestions.” The Cronbach's alpha coefficient for this scale was 0.892.

**Emotional exhaustion (EE):** This was measured using five items from the revised burnout scale, MBI-GS, developed by Li and Shi (2003). Example items include “Work makes me feel physically and mentally exhausted” and “Working all day long is very stressful for me.” The Cronbach's alpha coefficient for this scale was 0.905.

**Empowerment expectations (E):** since there is no standardized scale specifically for employee empowerment expectations, this variable was adapted from previous studies. Drawing on the methodological approaches of Humborstad and Kuvaas (2013) and Yin et al. (2022), we adapted the Leadership Empowerment Behavior Scale (Ahearne et al., 2005) by reframing the subject as “I expect” to assess employees' empowerment expectations. The psychometric properties of this adapted scale have been empirically validated in Chinese organizational contexts (Zhang and Bartol, 2010). Accordingly, the present study employs this established measurement approach to operationalize employees' empowerment expectations. The four dimensions of the scale are: “Enhancing meaning at work, facilitating participation in decision-making, conveying a message of high performance, and providing autonomy from bureaucracy.” The Cronbach's alpha coefficient for this scale was 0.875.

**Voice behavior (EB):** voice behavior in this study is defined as behavior that helps improve the effectiveness of decision-making and changes the status quo within the organization. The unidimensional voice behavior scale developed by LePine and Van Dyne (1998) was used to measure this variable. Six items, such as “Our team members often offer constructive ideas,” were included. The Cronbach's alpha coefficient for this scale was 0.925.

**Control variables:** control variables included employees' gender, age, education, team size, and firm size. These variables were used to more rigorously examine the relationships between shared leadership, perceived organizational status, emotional exhaustion, and other employee-related constructs.

## 4 Results

### 4.1 Common method variance

Regarding the analytical approach, we first performed Harman's single-factor test using SPSS 25.0 to examine potential common method bias in our measurement instruments. Subsequently, we employed AMOS 24.0 to conduct confirmatory factor analysis (CFA) for assessing the construct validity of the measurement model. Consistent with established practices in leadership research (Mui Hung Kee et al., 2020), we computed descriptive statistics and examined inter-variable correlations using SPSS 25.0. To test our hypothesized mediation, moderation, and conditional indirect effects, we conducted hierarchical regression analyses with the PROCESS macro (Version 4.0) for SPSS, applying bootstrapping procedures with 5,000 resamples to generate bias-corrected confidence intervals.

Utilizing AMOS24.0, a confirmatory factor analysis was undertaken on variables including SL, Perceived Organizational Status, Emotional Exhaustion, Empowerment Expectations, and Employee Voice Behavior in Table 1. As see in Table 1 the hypothesized five-factor model exhibited the most robust fit (χ2/*df* = 1.028, CFI = 0.999, TLI = 0.999, RMSEA = 0.027), outperforming other models including one-factor model (Δχ2 = 11,099.44, Δ*df* = 18, *p* < 0.001), and demonstrating sound discriminant validity for each factor examined.

**Table 1:** Confirmatory factor analysis result.

**Model**	**χ^2^**	***df***	**χ^2^*/df***	**RMSEA**	**CSI**	**TLI**	**SRMR**
Five-factor model	909.145	884	1.028	0.007	0.999	0.999	0.027
Four-factor model	2,432.419	888	2.739	0.053	0.916	0.911	0.089
Three-factor model	4,094.893	891	4.596	0.076	0.826	0.815	0.134
Two-factor model	4,391.157	893	4.917	0.079	0.810	0.799	0.142
One-way model	11,918.585	902	13.214	0.140	0.401	0.372	0.137

N, 624; SL, shared leadership; POR, perceived organizational status; EE, emotional exhaustion; E, empowerment expectations; VB, employee voice behavior. Five factor model = SL, POR, EE, E, VB, Four factor model = SL, POR + EE, E, VB, Three factor model = SL, POR + EE + VB, E, Two factor model = SL + E, POR + EE + VB, one-factor model = SL + POR + EE + E + VB.

This research relied on self-reported data, which may introduce biases such as social desirability bias or recall bias. To mitigate these potential biases, we employed anonymous survey methods, assigning each participant a unique identifier across the two time points to ensure anonymity and match responses over time. Furthermore, we utilized scales with high reliability and validity from top journals to further reduce potential common method biases arising from self-reporting. Previous research indicated that the common method bias is not a major concern if the result of Harman single-factor test were below 40% (Podsakoff et al., 2003). This study's Harman single-factor test indicated that the cumulative variance explanation of the first factor in this research is 28.87%, below the rule of thumb threshold of 40%, indicated that common method bias is not a major concern in this study.

### 4.2 Descriptive statistics

As shown in Table 2, SL is significantly positively correlated with POS (*r* = 0.523, *p* < 0.01), and POS is positively related to employees' voice behavior (*r* = 0.528, *p* < 0.01). Furthermore, SLP is significantly positively correlated with emotional exhaustion (*r* = 0.580, *p* < 0.01), while emotional exhaustion is negatively correlated with employees' voice behavior (*r* = −0.043). These results provide preliminary support for Hypotheses 1–4 of this study.

**Table 2:** Means, standard deviations, and correlations between variable.

**Variant**	**1**	**2**	**3**	**4**	**5**	**6**	**7**	**8**	**9**	**10**	**11**	**12**
1.Gender	1											
2. Age	−0.056	1										
3. Education	−0.038	0.030	1									
4. Type of industry	0.042	−0.021	−0.010	1								
5. Type of business	0.078	0.057	0.007	−0.044	1							
6. Enterprise size	−0.001	−0.009	−0.019	−0.105^**^	−0.060	1						
7. Team size	−0.016	0.008	0.032	0.062	−0.139^***^	0.135^**^	1					
8. Shared leadership	0.033	0.068	0.033	0.011	−0.060	0.131^**^	0.360^***^	1				
9. Perceived organizational status	−0.013	0.029	−0.027	0.045	−0.074	0.107^**^	0.236^***^	0.523^***^	1			
10. Emotional exhaustion	0.056	0.102^*^	0.054	−0.012	−0.030	0.168^***^	0.292^***^	0.580^***^	0.286^***^	1		
11. Empowerment expectations	−0.007	0.062	0.045	−0.038	−0.073	0.150^***^	0.166^***^	0.338^***^	0.259^***^	0.269^***^	1	
12 Voice behavior	0.133	0.073	0.025	−0.032	0.026	−0.003	0.301^*^	0.350^**^	0.528^**^	−0.043	0.175^**^	1
13 M	1.46	2.96	2.49	2.24	2.36	2.45	2.97	3.40	3.48	3.54	3.37	3.39
14 SD	0.50	1.02	0.91	1.19	0.78	1.30	1.23	0.87	1.02	0.97	0.81	0.99

*N* = *624*, ^*^(p<*0.05)*, ^**^(p<*0.01)*, ^***^(p<*0.01)*.

### 4.3 Hypothesis testing

To examine the proposed mediation model, we employed the PROCESS macro developed by Hayes (2012), which has been widely validated for testing mediation and moderation effects in psychological research. Consistent with our theoretical framework, we utilized PROCESS Model 4 to analyze the mediation effects, and PROCESS Model 7 to examine the moderation effects. All analyses incorporated bias-corrected bootstrapping with 5,000 resamples to generate robust 95% confidence intervals (CI: confidence intervals) for the indirect effects, following current best practices in statistical mediation analysis. The outcomes of our regression analysis confirm that the direct effect of SL on employee voice behavior is indeed positive, with an effect size of 0.3496 (*p* < 0.01, 95% CI [0.3142, 0.4822]).

As show in Table 3, Hypothesis 1 proposed that SL have a positive effect on POR results of **model 1** indicate that SL positively contribute to a higher level of POR (β = 0.5228, *p* < 0.001, 95% CI = [0.5294,0.6854]), supported hypothesis 1.POR is positive related to employees voice behavior (**Model 3**, β = 0.4578, *p* < 0.001, 95% CI = [0.3858,0.5297]), and showed in Table 4 the positive mediating effect of POR on the link between SL and employee voice behavior was also confirmed (indirect effect = 0.2441, 95% CI = [0.2225, 0.3380]), supported hypothesis 2. Hypothesis 3 believed that SL may lead to employee emotional exhaustion. Results of **model 2** indicate that SL is positively related to employee emotional exhaustion (**Model 2**, β = 0.5802, *p* < 0.001, 95% CI = [0.5732,0.7152]), supported hypothesis 3. **Model 3** showed when added emotional exhaustion as another mediator that emotional exhaustion is negative related to employees voice behavior (**Model 3**, β = −0.3673, *p* < 0.001, 95% CI = [−0.4460, −0.2886]), and the negative mediating effect of emotional exhaustion on the link between SL and employee voice behavior was also confirmed (indirect effect = −0.2078, 95% CI =[−0.2899, −0.1879]), supported hypothesis 4.

**Table 3:** Regression results for main, mediation effects.

**Predictor**	**Model1: POR**				**Model 2: EE**				**MODEL 2: VB**			
	β	***SE***	**LLCI**	**ULCI**	β	***SE***	**LLCI**	**ULCI**	β	***SE***	**LLCI**	**ULCI**
SL	0.5228^***^	0.397	0.5294	0.6854	0.5802^***^	0.363	0.5732	0.7152	0.3568^***^	0.501	0.2585	0.4551
POR									0.4578	0.366	0.3858	0.5297
EE									−0.3673	0.401	−0.4460	−0.2886
R2	0.2733				0.3366				0.3717			
F	233.9658				315.5793				12.2433			

^***^(*p* < 0.01).

**Table 4:** Decomposition of total, direct, and mediation effects.

**Path**	**Std. Coeff**	**SE**	**t**	**p**	**95% Bootstrap CI**
SL → VB	0.3496	0.0428	9.3072	< 0.001	[0.3142, 0.4822]
SL → VB (controlling POR, EE)	0.3133	0.0501	7.1292	< 0.001	[0.2585, 0.4551]
**Indirect effects**					
SL → POR → EE	0.2441	0.0294	–		[0.2225, 0.3380]
SL → EE → VB	−0.2078	0.0261	-		[−0.2899, −0.1879]

**Figure 2:**
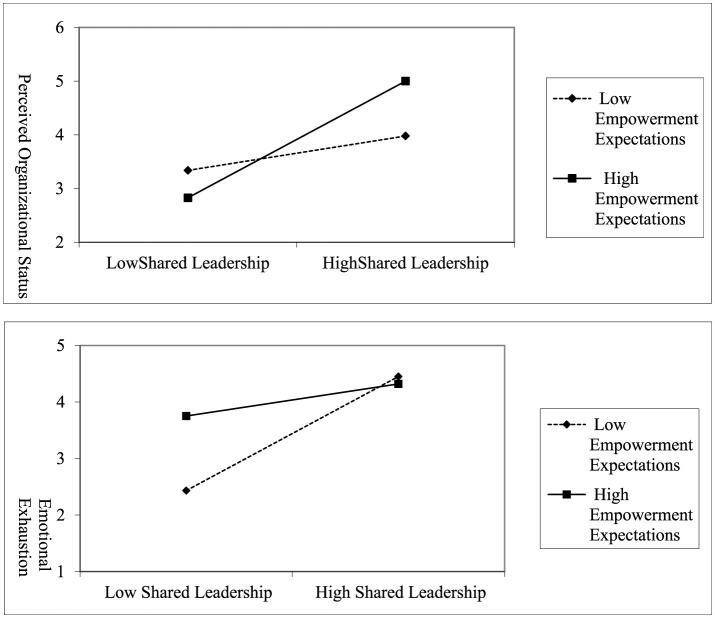
Moderating effect.

This study investigated the conditional influence of Empowerment Expectations on the dynamics between SL and employee voice behavior. As show in Table 4, the analysis unveiled that a fusion of SL and high Empowerment Expectations markedly enhanced POR, denoted by a significant moderation effect (moderation effect = 0.3210, β = 0.6222, *p* < 0.001, 95% CI = [0.5409, 0.7036]), suggesting that a higher level of Empowerment Expectations amplifies the beneficial link between SL and POR. As depicted in Figure 2, the facilitative impact of SL on POR was notably pronounced at an elevated level of empowerment expectations (1 SD above the mean, β = 0.8811, 95% CI = [0.7584, 1.0038], *p* < 0.001), While the effect remains significant at low Empowerment Expectations, its magnitude is substantially smaller compared to high empowerment expectations (1 SD below the mean, β = 0.3634, 95%CI = [0.2621, 0.4648], *p* < 0.001), Hypothesis 5 garnered empirical support.

As show in Table 5, Hypothesis 6 posited a negative moderation by Empowerment Expectations on the link between SL and employee voice behavior. The interaction between SL and Empowerment Expectations was found to significantly reduce employee emotional exhaustion (moderation effect = −0.2807, β = 0.5688, *p* < 0.001, 95% CI = [−0.3692, −0.1923]). As depicted in Figure 2, the impact of SL on emotional exhaustion was not pronounced at an elevated level of empowerment expectations (1 SD above the mean, β = 0.3424, 95% CI = [0.2299, 0.4549], *p* < 0.001) yet this association was strengthen when empowerment expectations was diminished (1 SD below the mean, β = 0.7952, 95%CI = [0.7023, 0.8881], *p* < 0.001), supported hypothesis 6.

**Table 5:** Analysis of moderating effects.

**MODEL5 (POR)**						**MODEL6 (EE)**				
	***Coeff***	***SE***	***p***	**LLCI**	**ULCI**	***Coeff***	***SE***	***p***	**LLCI**	**ULCI**
SL	0.6222	0.0414	0	0.5409	0.7036	0.5688	0.038	0	0.4943	0.6434
E	0.1306	0.0441	0.0032	0.044	0.2172	0.0867	0.0404	0.0324	0.0073	0.1661
Int_1	0.3210	0.0491	0	0.2245	0.4175	−0.2807	0.045	0	−0.3692	−0.1923
R^2^	0.327					0.381				
F	100.5					127.4				

**Figure 3:**
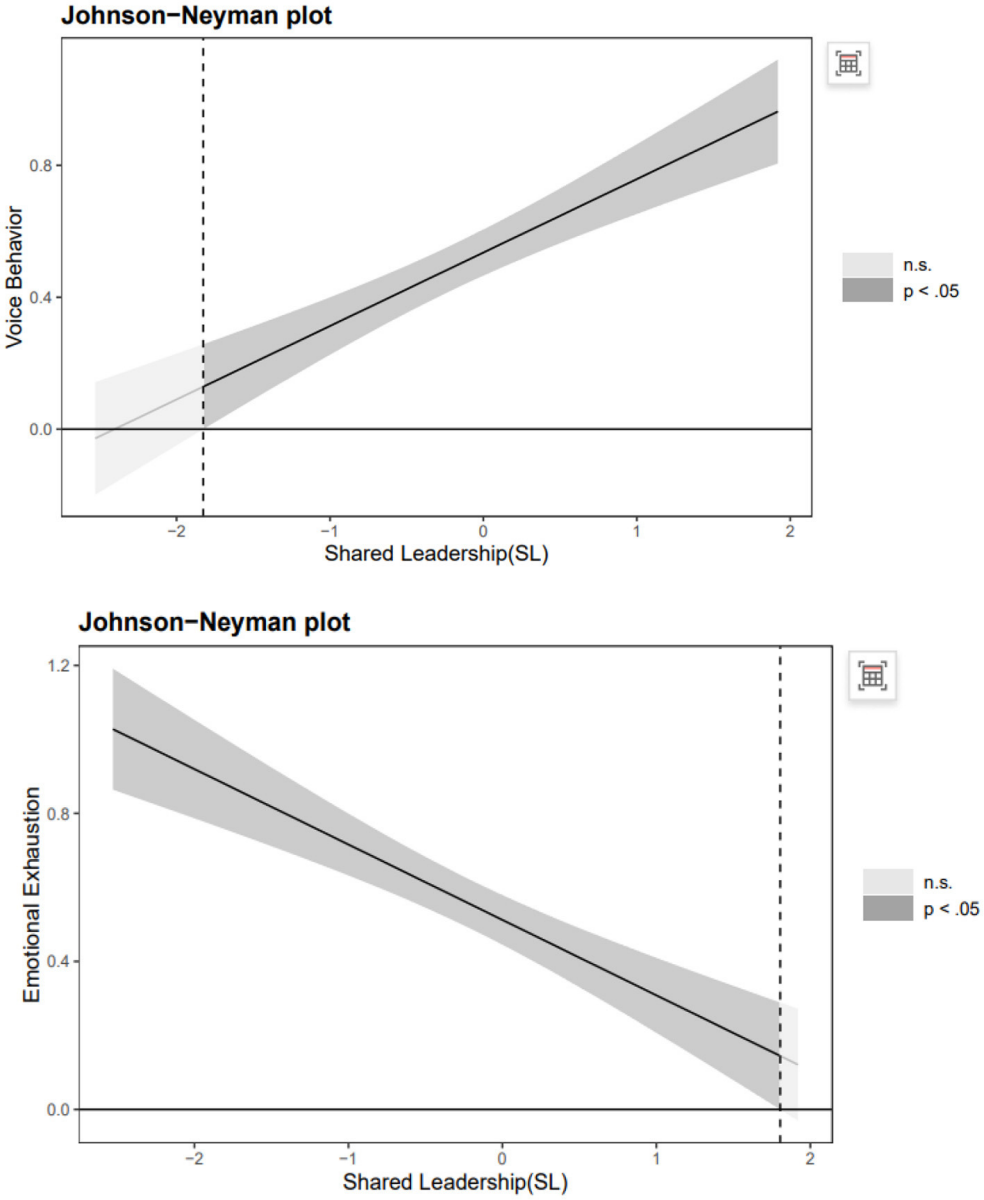
Adjustment effect simple slope plot.

When under a high level of Empowerment Expectations, the result showed a significantly positive conditional indirect effect of SL on employees voice behavior through POR(1 SD above the mean, indirect effect =0.4033, 95%CI=[0.3286, 0.4813]), however it was not significantly at a lower level of empowerment expectations (1 SD below the mean, indirect effect =0.1664, 95%CI=[0.1098, 0.2282]). As shown in Figure 3, the moderated mediation index of the POR was 0.1469 (95% CI = [0.1022, 0.1949]). Meanwhile, the conditional indirect effect of emotional exhaustion was negatively and significant in the full moderated mediation model under a lower level of Empowerment Expectations (1 SD below the mean, indirect effect = −0.2921, 95%CI = [−0.3579, −0.2277]), whereas it was not significant under a higher level of empowerment expectations (1 SD above the mean, indirect effect = −0.1258, 95%CI = [−0.1756, −0.0802]). The moderated mediation index of emotional exhaustion was 0.1031 (95% CI = [0.0697, 0.1397]). As shown in Figure 3, When ZE outside the interval [−3.42, −1.83], the slope of is p < 0.05.Whem ZE is outside the interval [1.80, 3.82], the slope of ZSL is *p* < 0.05. Hypothesis 7 was supported.

## 5 Discussion

Based on the Resource Conservation Theory and the Empowerment Slavery Model, this study examines the “double-edged sword” effects of shared leadership on employees' voice behaviors, the mediating roles of perceived organizational status and emotional exhaustion, and the moderating role of employees' empowerment expectations. The results reveal that the shared leadership model influences employee voice behavior, with the effects and underlying mechanisms varying based on employees' perceptions of the model. Specifically, employees who perceive themselves as holding an important position in the organization due to power sharing within the shared leadership model are more likely to advocate for the organization. In other words, shared leadership positively influences employees' voice behaviors through the mediating effect of perceived organizational status. Conversely, employees who experience work pressure and emotional exhaustion due to power rotation within the shared leadership model tend to inhibit their voice behaviors. Thus, shared leadership negatively affects employees' voice behaviors through the mediating effect of emotional exhaustion. Furthermore, employee empowerment expectations moderate the relationship between the shared leadership model and employees' voice behaviors. Specifically, employee empowerment expectations positively moderate the indirect effect of the shared leadership model on voice behaviors via perceived organizational status, while negatively moderating the indirect effect of shared leadership on voice behaviors via emotional exhaustion.

### 5.1 Theoretical implications

First, most previous studies have examined the facilitating or inhibiting effects of shared leadership from a single perspective, often overlooking the potential “double-edged sword” effect on employee voice behavior. Based on resource conservation theory and empowerment slavery theory, this study addresses the controversial issue of whether shared leadership can simultaneously promote and inhibit employees' constructive behaviors within the same framework. The findings confirm that the shared leadership model can both foster and hinder employees' constructive behaviors, effectively addressing previous disagreements and contradictions in the literature. This provides a theoretical foundation for a comprehensive understanding of the effectiveness, complexity, and bi-directionality of shared leadership.

Second, the impact of a leadership model is not solely determined by the team environment and leadership characteristics but also by subordinates' perceptions of the leadership model, which is a perceptual process. Employees' subjective perceptions of the leadership model can directly influence their work attitudes and behavioral outcomes. Particularly in the collectivist organizational context of China, employees tend to categorize themselves based on their perceptions of organizational status, which then induces varying behaviors. Drawing on empowerment-slavery theory, this study incorporates employees' subjective perceptions into the research framework, uncovering the “black box” of the mechanism between shared leadership and employee voice behaviors. Specifically, it highlights how employees' perceptions of organizational status and emotional depletion, triggered by the empowerment and slavery dimensions of the shared leadership model, affect voice behavior. This research expands the intermediary mechanisms through which shared leadership influences employee behavior, broadening the applicability of empowerment-slavery theory and offering new insights into the relationship between shared leadership and employee voice.

Third, while previous studies have primarily focused on shared leadership from the perspective of empowerment, there has been less emphasis on the dual perspectives of power-sharing and subordinate expectations, as well as on the effectiveness of the shared leadership model. This study focuses on how varying levels of employee empowerment expectations differentially impact shared leadership outcomes. By constructing a mediated model, we argue that employee empowerment expectations are critical to unlocking the effectiveness of shared leadership, thereby refining the boundary conditions for its success. The findings suggest that organizations can adopt the shared leadership model more rationally and scientifically, enabling them to garner more constructive advice from employees.

### 5.2 Management insights

First, this study highlights a critical phenomenon: influenced by Confucian hierarchical culture, hierarchical leadership remains deeply entrenched in Chinese enterprise management. Breaking through hierarchical norms to achieve genuine power sharing in team management is challenging. Many companies, under the guise of “shared leadership,” delegate tasks that should be collaboratively completed by the team to a select few high-performing employees. In essence, while the shared leadership model distributes more workloads and responsibilities among members, decision-making authority continues to reside with formal leaders. This imbalance inevitably increases work pressure and emotional exhaustion for some employees, ultimately undermining organizational participation.

Second, Base on the leader-member exchange (LMX) theory (Grean and Uhl-Bien, 1995), high-quality hierarchical relationships may mitigate the negative effects of shared leadership. To support employees under this model, organizations could implement interventions such as regular psychological counseling, company-sponsored social activities, formal grievance channels, and optimized job design. These measures may alleviate role stress and prevent the adverse consequences of resource depletion on employee wellbeing. Therefore, the implementation of shared leadership in China should be adapted dialectically to align with local management practices.

Third, the effectiveness of the shared leadership model also depends on the level of employees' empowerment expectations. Managers must address these varying empowerment expectations to mobilize and stimulate employee motivation, thus promoting constructive behaviors. If there are many employees with high empowerment expectations in the team, leaders can leverage the shared leadership model to engage and empower these employees. Conversely, if there are more employees with low empowerment expectations, leaders should adopt a nurturing approach and apply the shared leadership model cautiously, to mitigate the negative attitudes that may arise from resistance to empowerment behaviors.

### 5.3 Shortcomings and prospects

Although this study provides valuable theoretical insights and empirical evidence, there are still some limitations. First, while the data in this study were collected from different time periods, the research relied solely on data from Dalian enterprises due to limited social resources. Additionally, the data were based on the self-reported judgments of leaders or employees, which could not fully eliminate the influence of common method bias. Future research could improve the accuracy of results by incorporating data from multiple sources, time points, and subjects.

Second, this study defines employee advising behavior as facilitative advising that proposes innovative ideas to solve current organizational problems, focusing on the frequency and number of advising behaviors. Future studies could differentiate between various types of advising behavior and explore the mechanisms through which the shared leadership model affects different types of advising behavior, as well as persistent advising behavior over time.

Finally, the dynamics of employee empowerment expectations is a topic of growing importance in organizational behavior research. This study only measured employee empowerment expectations at a single point in time. Future research should focus on the dynamic changes in employee empowerment expectations by conducting longitudinal case studies with multiple measurements, to investigate how these changes affect the effectiveness of the shared leadership model over time.

## Data Availability

The original contributions presented in the study are included in the article/supplementary material, further inquiries can be directed to the corresponding author.
